# Overexpression of *OsMYB48-1*, a Novel MYB-Related Transcription Factor, Enhances Drought and Salinity Tolerance in Rice

**DOI:** 10.1371/journal.pone.0092913

**Published:** 2014-03-25

**Authors:** Haiyan Xiong, Jinjie Li, Pengli Liu, Junzhi Duan, Yan Zhao, Xiao Guo, Yang Li, Hongliang Zhang, Jauhar Ali, Zichao Li

**Affiliations:** 1 Key Lab of Crop Heterosis and Utilization of Ministry of Education and Beijing Key Lab of Crop Genetic Improvement, China Agricultural University, Beijing, People’s Republic of China; 2 International Rice Research Institute, Metro Manila, Philippines; Purdue University, United States of America

## Abstract

MYB-type transcription factors (TFs) play essential roles in plant growth, development and respond to environmental stresses. Role of MYB-related TFs of rice in drought stress tolerance is not well documented. Here, we report the isolation and characterization of a novel MYB-related TF, *OsMYB48-1*, of rice. Expression of *OsMYB48-1* was strongly induced by polyethylene glycol (PEG), abscisic acid (ABA), H_2_O_2_, and dehydration, while being slightly induced by high salinity and cold treatment. The OsMYB48-1 protein was localized in the nucleus with transactivation activity at the C terminus. Overexpression of *OsMYB48-1* in rice significantly improved tolerance to simulated drought and salinity stresses caused by mannitol, PEG, and NaCl, respectively, and drought stress was caused by drying the soil. In contrast to wild type plants, the overexpression lines exhibited reduced rate of water loss, lower malondialdehyde (MDA) content and higher proline content under stress conditions. Moreover, overexpression plants were hypersensitive to ABA at both germination and post-germination stages and accumulated more endogenous ABA under drought stress conditions. Further studies demonstrated that overexpression of *OsMYB48-1* could regulate the expression of some ABA biosynthesis genes (*OsNCED4*, *OsNCED5*), early signaling genes (*OsPP2C68*, *OSRK1*) and late responsive genes (*RAB21*, *OsLEA3*, *RAB16C* and *RAB16D*) under drought stress conditions. Collectively, these results suggested that *OsMYB48-1* functions as a novel MYB-related TF which plays a positive role in drought and salinity tolerance by regulating stress-induced ABA synthesis.

## Introduction

Crop plants are frequently exposed to variable abiotic stresses. Among them, drought is a major stress factor for crop growth and production. During the long evolutionary history, plants have evolved various mechanisms to survive in the stress environments, such as changes in internal molecules, cellular and physiological processes [1]. Abscisic acid (ABA) is such an essential phytohormone that regulates various aspects of plant growth and development in response to the abiotic stresses [2]. ABA content increases dramatically when plants are exposed to abiotic stresses such as drought and salinity that triggers the expression of many genes encoding various proteins important for biochemical and physiological processes [3]–[5]. These proteins help plants be tolerant to the stresses that includes late embryogenesis abundant (LEA) proteins, enzymes involved in osmo-protectant synthesis, protection proteins for plants from oxidative damage, and signaling proteins, such as the transcription factors (TFs) [2].

TFs like MYB, AP2/ERF, NAC, bZIP, and WRKY families act as the early responders to environmental signals and trigger the expression of stress-induced genes that are important for plants to be tolerant to abiotic stresses [6]–[8]. In recent years, many researchers have demonstrated the utilization of TFs in the engineering of crop plants as a powerful approach for enhanced tolerance against abiotic stresses. For instance, overexpression of *AP37*, *OsNAC10*, *OsbZIP23* and *OsbZIP46* in rice can significantly improve the tolerance to drought and high-salinity stresses [9]–[12].

MYB is a large TF family in plants. There are over 198 MYB genes in *Arabidopsis* and over 183 members in rice [13]. According to the number of imperfect repeats of the SANT (for SWI3, ADA2, N-CoR, and TFIIIB) (50–53 amino acids) in DNA-binding domains, plant MYB proteins can be classified into three major subfamilies: the MYB-related group (one single SANT domain), the R2R3-type group (two SANT domains) and R1R2R3-MYB group (three SANT domains) [13], [14]. Over the past decade, it has been reported that the most of MYB genes, involve in response to diverse abiotic stress, belong to the R2R3-type group. For instance, *AtMYB2* functions in the ABA-mediated drought stress response in *Arabidopsis* [15]. *AtMYB102* is a key regulatory component in responses of *Arabidopsis* to wounding, osmotic stress, salinity stress and ABA application [16]. In addition, *AtMYB96* modulates ABA signaling in response to abiotic stresses in *Arabidopsis* [17]. In rice, *OsMYB4* was earlier reported to play a positive role in transgenic *Arabidopsis*, tomato, and apple to cold and drought tolerance [18]–[20]. *OsMYB55* was shown to be involved in tolerance to high temperature through enhanced amino acid metabolism [21]. In a recent study, molecular characteristic features of *OsMYB2* have clearly been indicating its regulatory role in salt, cold, dehydration tolerance in rice [22].

Compared with R2R3-type MYB genes, few studies of the MYB-related group genes in abiotic stress response have been reported in plants. In potato, the single MYB domain TF *StMYB1R-1* has been shown to involve in drought tolerance through activation of drought-related genes [23]. Overexpression of a single-repeat MYB TF *AmMYB1* from grey mangrove confers salt tolerance in transgenic tobacco [24]. So far, MYBS3 is the only MYB-related protein in rice, which was reported to be involved in abiotic stresses, and it is essential for cold stress tolerance of rice [25]. However, little is known about the role of MYB-related TFs of rice in drought and salt stress tolerance.

Based on our cDNA microarray data published previously [26], we isolated a novel rice MYB-related type gene, *OsMYB48-1*. In this study, we found that the expression of *OsMYB48-1* was induced by various abiotic stresses. In order to carry out functional studies on *OsMYB48-1* gene and later on to apply it for genetic engineering of crops, firstly we have developed the transgenic rice overexpressing *OsMYB48-1.* Our study clearly indicated that many ABA-related genes were up-regulated in the *OsMYB48-1-*overexpression plants especially under drought stress and the transgenic plants had shown improved tolerance to drought and high salinity stress. All these findings will be utilized for the engineering crops with drought and salinity tolerance.

## Materials and Methods

### Plant Growth Conditions and Stress Treatments

To detect the transcription level of *OsMYB48-1* under various abiotic stresses and phytohormone treatment, seeds of Nipponbare (*O. sativa* L. ssp. japonica) were sterilized in 75% ethanol for 2 min and in 20% NaClO for 30 min, and washed with sterile water thoroughly. The sterilized seeds were germinated at 37°C for 2 d and grown hydroponically with Hoagland solution in growth chamber (28°C/25°C (day/night) with a 12 h photoperiod). Three-week-old seedlings were treated with stress including dehydration stress (the intact seedlings were exposed in the air without water supply), high osmotic pressure using 20% (w/v) polyethylene glycol (PEG) 6000 solution, salinity stress using 150 mM NaCl solution, cold exposure at 4°C, oxidative stress using 1 mM H_2_O_2_ solution, and hormone treatment using 100 mM ABA solution followed by sampling in a pre-determined time manner. Total RNA was extracted from leaves and used for further analysis.

### RNA Isolation and qRT-PCR Analysis

Total RNA was isolated from the rice leaves using RNAiso Plus (TaKaRa, Dalian, China) according to the manufacturer’s instructions. The DNase-treated RNA was reverse-transcribed into first-strand cDNA with M-MLV reverse transcriptase (TaKaRa, Dalian, China) according to the manufacturer’s instructions. A fivefold dilution of the resultant 1^st^ strand cDNA was used as template for PCR. Quantitative real-time PCR (qRT-PCR) was performed according to described previously [27]. The rice *Actin* gene was used as the endogenous control for data normalization. Each analysis was repeated at three times. All the primers were listed in Table S1.

### Sequence Analysis

The homolog genes of *OsMYB48-1* were searched through BLAST database (http://www.ncbi.nlm.nih.gov/BLAST/). The phylogenetic tree was constructed in MEGA5 software based on neighbor-joining method. Numbers indicate percentage values after 1000 replications. The multiple sequence alignment was performed with ClustalW. We searched the promoter sequence (2,000 bp upstream from the transcription start site) against the PLACE database (http://www.dna.affrc.go.jp/PLACE/signalscan.html) to detect the *cis*-acting elements for reveal the regulatory roles of *OsMYB48-1*.

### Plasmid Construction and Plant Transformation

To generate *OsMYB48-1* overexpression plants, the full-length coding region of *OsMYB48-1* was amplified from the first-strand cDNA of Nipponbare using gene-specific primer pairs and cloned into the *Asc* I and *Pac* I sites of binary expression vector PMDC32 under the control of double *Cauliflower Mosaic virus* (*CaMV*) *35S* promoter. The resultant construct was transformed into *Agrobacterium tumefaciens* EH105 and then transformed into Nipponbare by *Agrobacterium*-mediated transformation method [28]. The transgenic plants were selected in 1/2 MS medium containing 50 mg/L hygromycin (Roche, Germany).

To generate the RNA interference (RNAi) plants, the 462 bp cDNA sequence was amplified and cloned into the *Kpn* I and *Bam*H I and then *Spe* I and *Sac* I sites of the pTCK303 vector as described previously [29]. Plant transformation protocols were taken as described above.

### Subcellular Localization and Transactivation Assay

To determine its subcellular localization, the whole *OsMYB48-1* coding region without stop codon was amplified and sub-cloned into the binary vector PMDC83, to produce OsMYB48-1-GFP fusion construct driven by *CaMV 35S* promoter. The recombinant constructs and free GFP were introduced into onion (*Allium cepa*) epidermal cells, performed with a model PDS-1000/He Biolistic particle delivery system (Bio-Rad, CO, USA). After incubation at 25°C for 24 h, the green fluorescence signal was observed using a confocal laser scanning microscope LSM510 Meta (Carl Zeiss; http://www.zeiss.com/) with an argon laser excitation wavelength (488 nm).

The transactivation experiment was carried out according to the manual of Yeast Protocols Handbook (Clontech). The full length coding region and truncated fragments of *OsMYB48-1* generated by PCR amplification were fused in frame to the GAL4 DNA binding domain in the vector of pGBKT7 (Invitrogen). pGBKT7 was used as a negative control, while the pGBKT7-53 was used as a positive control. These constructs were transformed into Yeast strain AH109 by the Lithium acetate-method. The transformed yeast strains were screened on the selective medium plates without Tryptophan (SD/−Trp), and cultured at 30°C for 2 d. The PCR-verified positive strains were transferred to SD medium lacking Tryptophan/Histidine/Adenine (SD/−Trp/−His/−Ade) and cultured for 2 d, and the *in vivo* agar plate assay (x-α-gal in medium) was performed to analyze the transactivation activities.

### Evaluation of Transgenic Plants in Response to Various Stresses

The seeds of *OsMYB48-1* overexpression lines and the wild type (WT) were germinated in 1/2 MS medium for various stress evaluations. For osmotic and high salinity treatment, the 3 d seedlings of both transgenic and WT plants (10 plants each repeat, 3 repeats) were transplanted to normal 1/2 MS medium, and 1/2 MS medium containing 200 mM mannitol and 150 mM NaCl, respectively. After 10 d at 28°C/25°C (day/night) with a 12 h photoperiod, shoot height and fresh weight of each line and WT were measured.

For dehydration treatment, three-week-old seedlings of both the *OsMYB48-1* transgenic and WT plants were grown hydroponically in bottom removed 96-well plates using Hoagland solution containing 20% (w/v) PEG6000 for 3 d. Then, the stressed plants were recovered in normal Hoagland solution for 10 d. Survival rates of each line and WT were investigated.

For drought treatment, 20 seedlings of the transgenic and WT in each flowerpot at 140 mm diameter x 160 mm deep filled with well forest soil and vermiculite (1∶1) were grown for one month. After creating drought-stress by not watering for 7 days under outdoor conditions, the seedlings were followed by recovery with water supply for the next 10 days. Plants were regarded as survivals if there were green and healthy young leaves after re-watering treatment. Survival rate was calculated as the ratio of number of survived plants over the total number of treated plants in a flowerpot. The representative plants of the transgenic and WT were photographed before and after the drought treatments. Each stress test was repeated at three times.

### Water Loss Assay and Quantification of Malondialdehyde (MDA) and Proline

To detect the rate of water loss under dehydration conditions, the leaves of the transgenic and WT plants grown under normal growth conditions for three weeks were detached and weighed immediately as the initial weight. The samples were placed on a laboratory bench at room temperature and weighed at the pre-determined time intervals. The rate of water loss was calculated on the basis of the initial weight of the leaves [27]. Ten plants of each transgenic and WT line were used in this assay, and three replicates were made for each line.

The malondialdehyde (MDA) content was measured following the methods described by Duan *et al* [27]. Free proline content was determined according to the methods used by Bates *et al* [30] and *Song et al* [31].

### ABA Sensitivity Test

The sensitivity of seed germination to ABA was assayed in 1/2 MS medium with ABA. Seeds of the transgenic and WT were germinated in 1/2 MS medium (30 seeds each, 3 repeats) containing four concentrations of ABA (0, 2, 3 and 5 μM) and the germination rates were recorded after 7 d.

To test the ABA sensitivity at post-germination stage, the transgenic and WT were germinated in normal 1/2 MS medium plates. The seedlings (about 3 days, 10 plants each, and 3 repeats) were transferred to 1/2 MS medium containing 0 μM and 3 μM ABA. Plant height and fresh weight of each seedling were measured after 10 d of the ABA treatment in a greenhouse at 28/25°C (day/night) with a 12 h photoperiod.

### Endogenous ABA Content Assay

Fresh leaves (0.2 g) from both the normal and drought stressed (without water for 5 d) plants were ground in an ice-cooled mortar, and homogenized in 4 mL 80% methanol containing 1 mM butylated hydroxytoluence as an antioxidant. The extract was incubated at 4°C for 4 h and centrifuged at 4,000 g for 20 min. The supernatant was passed through a Sep-Pak C18 cartridge (Waters Corp. Milford, MA), and prepared for endogenous ABA determination using enzyme-linked immunosorbent assay (ELISA) as described previously [32]. Each data point was the average value of three replicates.

### Accession Numbers

Sequence data from this article can be found with the following accession numbers in the GenBank databases: *Actin* (AK100267), *NCED4* (AK11978), *NCED5* (AY838901), *OsPP2C68* (AK063334), *OSRK1* (AB125307), *RAB21* (AK121952), *LEA3* (AK119713), *RAB16C* (AK071366), *RAB16D* (AK109096), *P5CS1* (AK102633) and *P5CS2* (AK069425).

## Results

### Isolation and Characterization of *OsMYB48-1*

Based on our previous work of rice expression profiling under water stress using a cDNA microarray, an EST showing increased expression level after water stress was identified [26]. The EST showed homology to known MYB genes. Two transcripts that resulted from alternative splicing were identified from the Gramene database, designated as *OsMYB48-1* and *OsMYB48-2* (with locus name LOC_Os01g74410.2 and LOC_Os01g74410.1). OsMYB48-1 misses the first R region, and encodes a single MYB-like domain TF, while OsMYB48-2 encodes a R2R3 type MYB TF. Here, we first introduce the isolation and characterization of *OsMYB48-1*. The gene comprises 1583 bp nucleotides with a 633 bp open reading frame, 494 bp 5′ UTR, 456 bp 3′ UTR, and without intron. It encodes a putative protein of 211 amino acids with a calculated molecular mass of 24.0 kD and a pI of 6.7855. Amino acid analysis revealed that the OsMYB48-1 is a MYB-related type protein with one single conserved SANT domain at the N terminus between 45 and 90 amino acids.

The phylogenetic tree based on the amino acid sequences of plant MYB proteins indicated that the OsMYB48-1 amino acid sequence showed high similarity with other MYB proteins, including AtMYB48 and AtMYB59 in Arabidopsis, OsMYB146 and OsMYB154 in rice (Fig. S1), indicating the possibility of functional conservation between them.

Sequences of various putative stress response-related *cis*-acting elements were identified in the promoter region of *OsMYB48-1*, such as MYB, MYC recognition site, W-box element, RAV and GCC-box element, and so on. The predicted functions and frequencies of these *cis*-acting elements were summarized in Table S2. Such an enriched presence of stress response-related *cis*-acting elements may suggest a critical role of *OsMYB48-1* in stress tolerance.

### *OsMYB48-1* Expresses in Various Tissues and is Induced by Different Stresses

**Figure 1:**
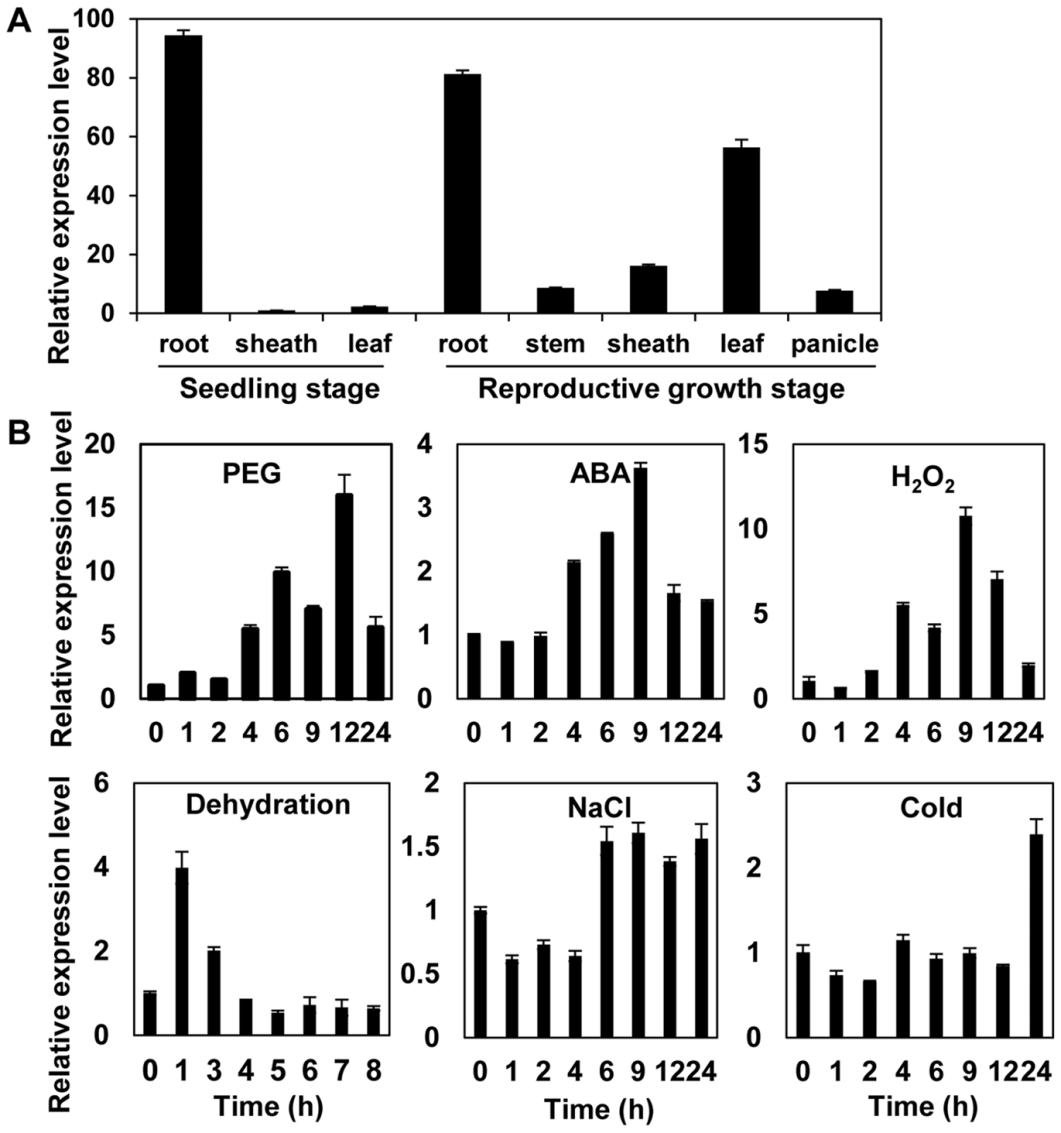
Expression analysis of the *OsMYB48-1* gene. (A) qRT-PCR analysis of the expression level of *OsMYB48-1* in different tissues of Nipponbare. (B) Expression patterns of *OsMYB48-1* under various stress treatments including PEG, ABA, H_2_O_2_, dehydration, NaCl, and cold. Error bars indicate standard error (SE) based on 3 replicates.

Tissue specificity may be associated with specific biological functions. We characterized the expression patterns of *OsMYB48-1* with qRT-PCR in various organs at seedling stage and productive stages. As shown in Figure 1A, *OsMYB48-1* was expressed in various tissues, including root, stem, sheath, leaf and panicle, but mainly expressed in roots at both seedling stage and reproductive stage, whereas was lowly expressed in sheath at seedling stage.

As described above, the promoter region of *OsMYB48-1* comprises of several stress response-related *cis*-elements. The results of inducible expression analysis showed that *OsMYB48-1* was strongly induced by PEG, H_2_O_2_, ABA, and dehydration, and slightly induced by NaCl and cold treatment (Fig. 1B). It suggested that *OsMYB48-1* may be involved in response to multiple abiotic stresses.

### Nucleus Localization and Transactivation Activity of *OsMYB48-1*

**Figure 2:**
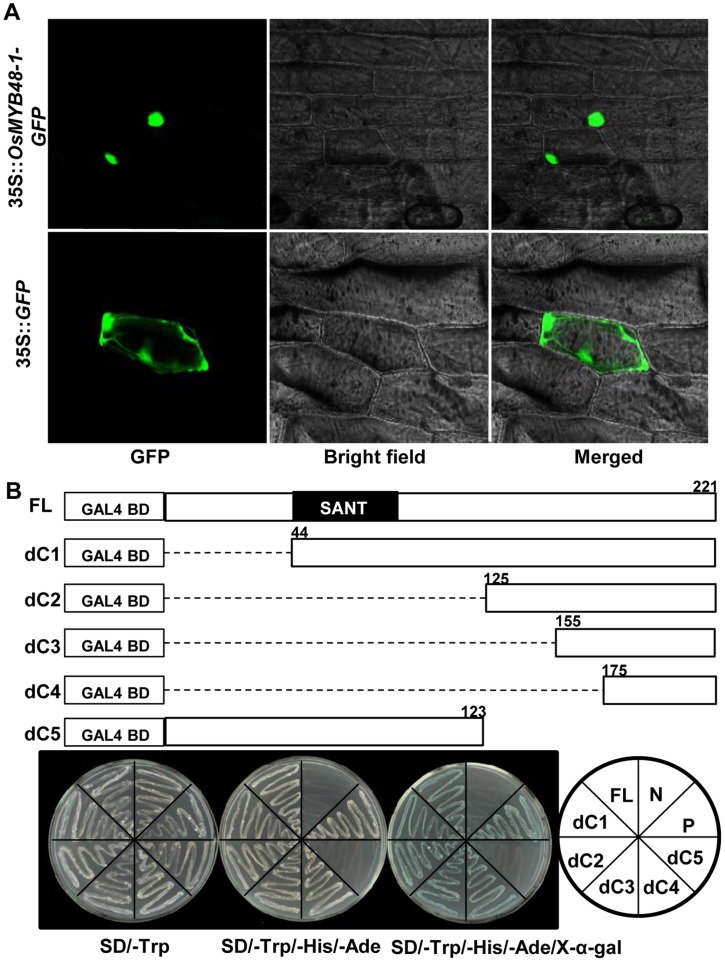
Subcellular localization and transactivation activity of OsMYB48-1. (A) The OsMYB48-1-GFP fusion protein was transiently expressed in onion epidermal cells and visualized by fluorescence microscopy. The upper panels show the localization of OsMYB48-1-GFP in onion cells in a transient assay, while bottom panels show the localization of GFP as a control. (B) Transactivation assay of truncated OsMYB48-1. Fusion proteins of the GAL4 DNA-binding domain and different portions of OsMYB48-1 were expressed in yeast strain AH109. FL indicates the full-length of OsMYB48-1; dC1 to dC5 indicate the mutated forms of OsMYB48-1 (nucleotide positions were labeled in the diagrams), respectively. P and N indicate the positive and negative control, respectively.

As predicted that *OsMYB48-1* is a TF, a nuclear localization signal was detected in OsMYB48-1-GFP transformed cell, while the free GFP showed ubiquitous distribution of signal in the whole cell (Fig. 2A), implying the role of OsMYB48-1 as a nuclear localized protein. To check if OsMYB48-1 has transactivation activity, the full length coding region and a series of truncated fragments of *OsMYB48-1* were constructed to the pGBKT7 vector and transformed into yeast. *In vivo* plate assay showed that OsMYB48-1 has transcriptional activity, and serial deletion in the N-terminal region from the start to the position of 174 aa did not affect the activation (Fig. 2B). These results confirmed that OsMYB48-1 is a transcription activator, and the C-terminal has transactivation activity.

### Overexpressing *OsMYB48-1* Enhances Drought and Salinity Tolerance

As described above, the expression of *OsMYB48-1* was induced by multiple abiotic stresses suggesting that *OsMYB48-1* may play a positive role in the regulation of stress tolerance. To confirm this, ten transgenic lines with overexpression of *OsMYB48-1* were obtained. We also obtained six RNAi transgenic lines, but the expression level of *OsMYB48-1* in all these lines showed no obvious decrease (Fig. S2). Therefore, just overexpression lines (T3, T6, and T10) with normal morphology (Fig. S3) were selected to further study the physiological function of *OsMYB48-1*.

**Figure 3:**
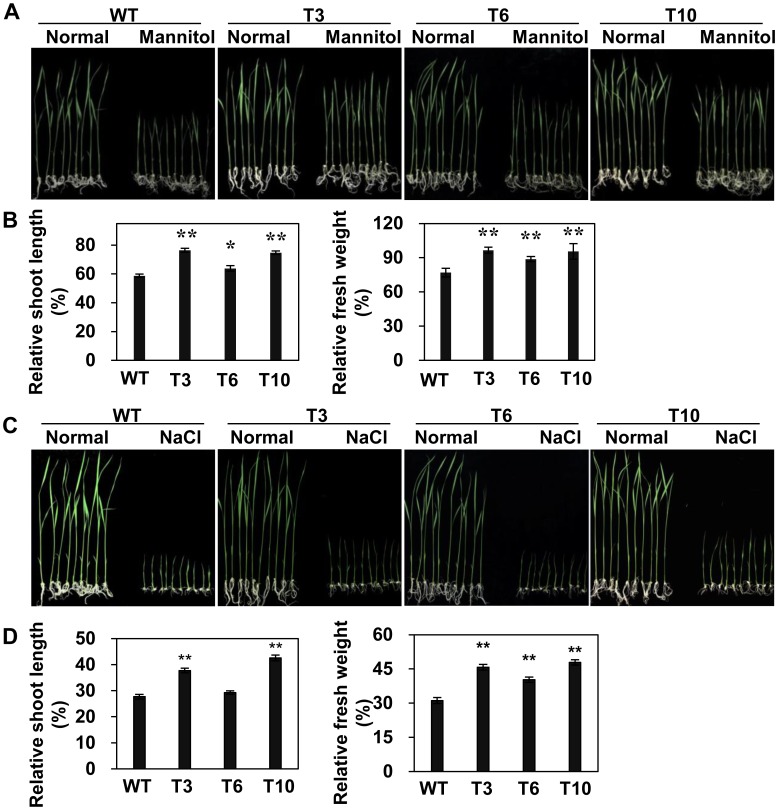
Osmotic and high salinity stress tolerance of *OsMYB48-1* overexpression plants. (A) Growing performance of *OsMYB48-1-*overexpression plants and WT on osmotic stress medium caused by 200 mM mannitol. (B) The relative shoot length and relative fresh weight of the transgenic and WT plants testing in (A) were compared. (C) Growing performance of transgenic and WT plants on high salinity stress medium caused by 150 mM NaCl. (D) The relative shoot length and relative fresh weight of the transgenic and WT plants testing in (C) were compared. Values are means ± SE (n = 30). * and ** indicate significant difference at P<0.05 and P<0.01 probability, respectively.

Under high osmotic stress conditions caused by mannitol treatment, the transgenic plants showed less growth inhibition than WT (Fig. 3A). Overexpression lines showed significantly lesser suppression of relative shoot growth (63.83% to 76.45% of normal growth) than WT (only about 58.72% of the normal growth), and also lesser suppression of relative fresh weight (88.8% to 96.57% of normal growth) than WT (only about 76.93% of the normal growth) (Fig. 3B).

Considering the cross-talk between the drought and high salinity response pathway [33], the performance of *OsMYB48-1* overexpression lines on high salinity stress was also determined. The results revealed that the transgenic plants exhibited lesser growth inhibition than WT (Fig. 3C), resulting from the significantly higher relative shoot growth (29.25%–42.57%) and relative plant fresh weight (40.25%–47.87%) of the overexpression lines than that of WT (27.67% and 31.13%, respectively) (Fig. 3D).

**Figure 4:**
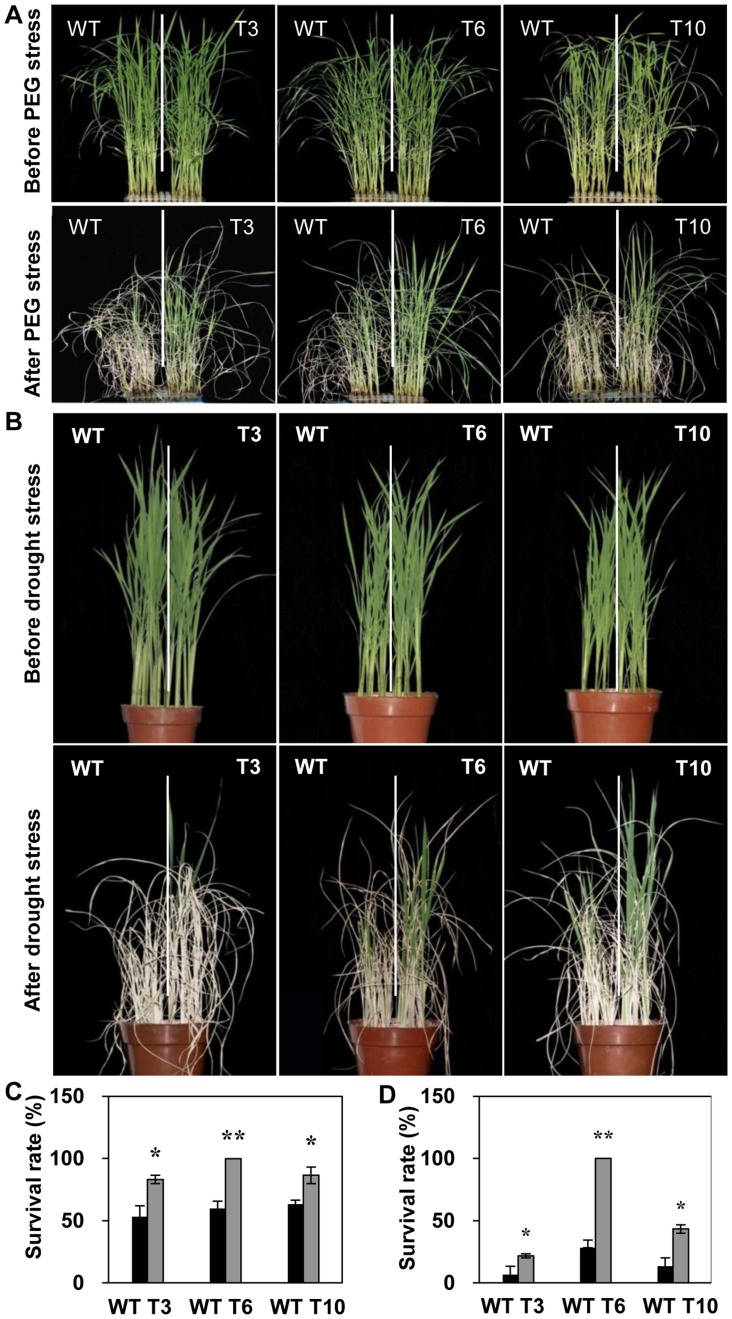
Drought stress tolerance of *OsMYB48-1* overexpression plants. (A) Mimic physiological dehydration stress tolerance assay of the *OsMYB48-1* transgenic and WT plants under 20% PEG treatment. (B) Performance of *OsMYB48-1* transgenic and WT plants subjected to soil drought stress without water for 7 d and then recovered for 10 d. (C) Survival rates of transgenic and WT plants testing in (A). Values are means ± SE (n = 3). (D) Survival rates of the transgenic and WT plants testing in (B). Values are means ± SE (n = 3). * and ** indicate significant difference at P<0.05 and P<0.01 probability, respectively.

Under PEG treatment that mimics the physiological dehydration stress condition, the transgenic plants had shown higher survival rate in the range of 83.33 to 100% as compared to WT with only 53.33% to 63.33% (Fig. 4A, C).

Likewise, under soil drought stress conditions caused by not watering, the transgenic plants had showed significantly higher survival rate in the range of 21.67 to 100% as compared to WT plants with 6.67 to 28.33%. (Fig. 4B, D).

Overexpression of *OsMYB48-1* showed significant increase for drought and salinity tolerance in rice based on the above findings.

### Physiological and Biochemical Changes in *OsMYB48-1* Overexpression Plants

**Figure 5:**
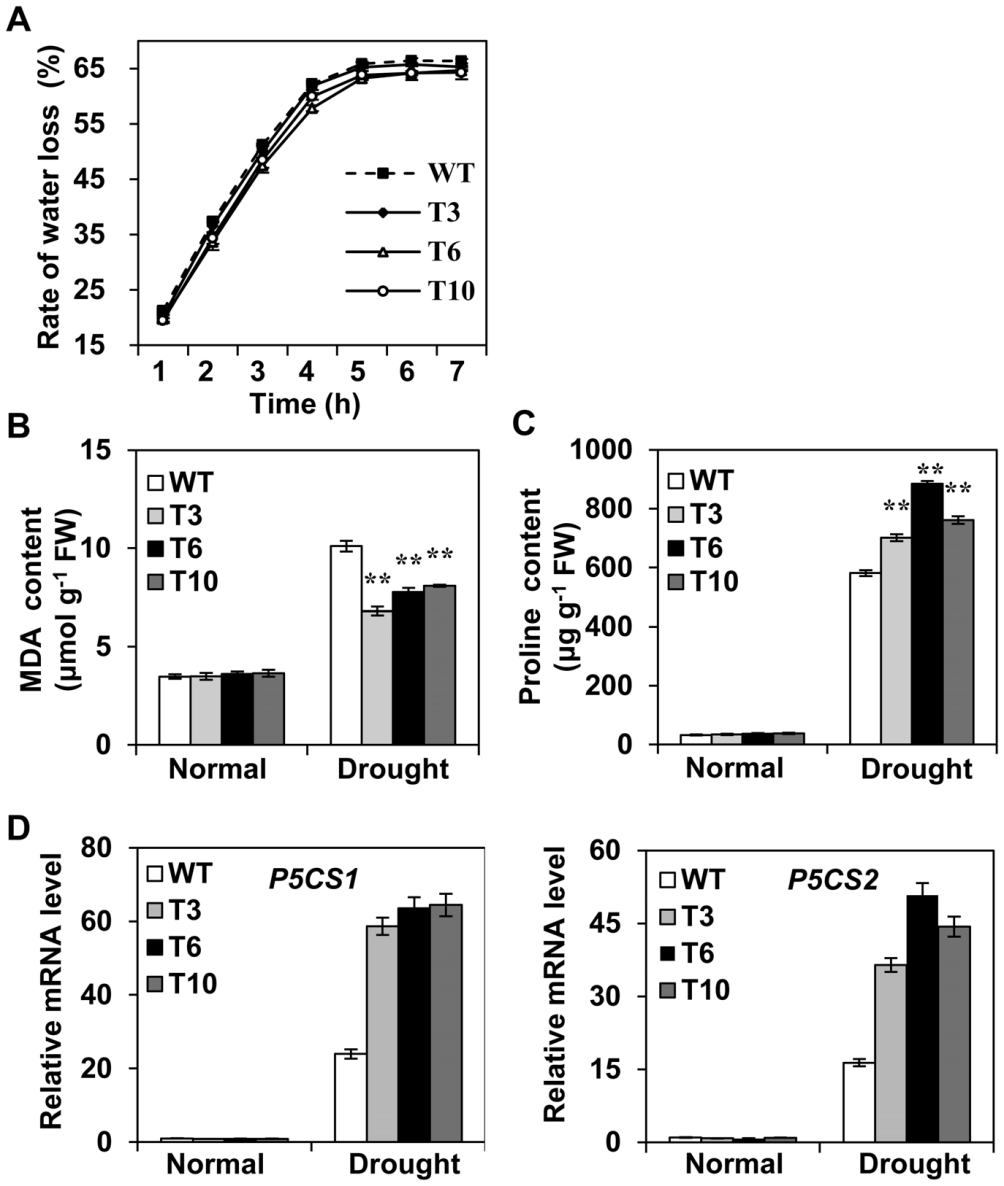
Physiological and biochemical changes in *OsMYB48-1* overexpression plants. (A) Rate of water loss in detached leaves cut from the *OsMYB48-1* transgenic and WT plants under normal conditions. Values are means ± SE (n = 10). (B) MDA contents. (C) Free proline contents. The MDA and free proline contents were measured in the leaves of two month old plants under natural condition and separately after creating drought treatment conditions by withholding water to plants for 5 d. Values are means ± SE (n = 4). FW, fresh weight. * and ** indicate significant difference at P<0.05 and P<0.01. (D) Expression of drought stress responsive genes in *OsMYB48-1* transgenic plants and WT. The qRT-PCR analysis was performed with total RNA from *OsMYB48-1* transgenic plants and WT under normal or drought stress treatment (withholding water for 5 d). Values are means ± SE (n = 3). * and ** indicate significant difference at P<0.05 and P<0.01 probability, respectively.

Rate of water loss in detached leaves is an important trait to detect drought tolerance and the transgenic plants showed a lower rate of water loss as compared to WT (Fig. 5A), indicating *OsMYB48-1* gene’s possible role in reducing water loss especially under drought stress conditions.

Under abiotic stressed conditions, ROS gets accumulated in plant tissues leading to oxidative damage, as indicated by the membrane lipid peroxidation. Malondialdehyde (MDA) is a lipid peroxidation product and serves as a biomarker for membrane lipid peroxidation. As shown in Figure 5B, drought stress conditions enhanced MDA production in the leaves of both transgenic and WT plants, however it was significantly lower in the transgenic overexpression plants (6.8–8.1 μg/g) as compared to WT (10.11 μg/g). These results suggested that transgenic lines overexpressing *OsMYB48-1* exhibited less oxidative damage under drought stress conditions.

Accumulation of proline to counteract the effects of osmotic stress is a common adaptive mechanism for plant responses to stress conditions [22]. Proline content increased under stressed conditions in both transgenic plants and WT, however, it was significantly higher in transgenic plants (701-884 μg/g) as compared to WT (581 μg/g) (Fig. 5C). Consequently, we detected the transcription levels of two proline biosynthesis genes, *OsP5CS1* and *OsP5CS2* in transgenic plants and WT. As shown in Figure 5D, the expression levels showed non-significant differences between the transgenic plants and WT under normal growth conditions. However, it was significantly higher in the transgenic plants than WT under drought stress conditions. These results clearly indicated that the overexpression of *OsMYB48-1* could increase the production of proline by regulating proline biosynthesis genes in rice especially under drought stress conditions.

### Increased ABA Sensitivity of *OsMYB48-1* Overexpression Plants

**Figure 6:**
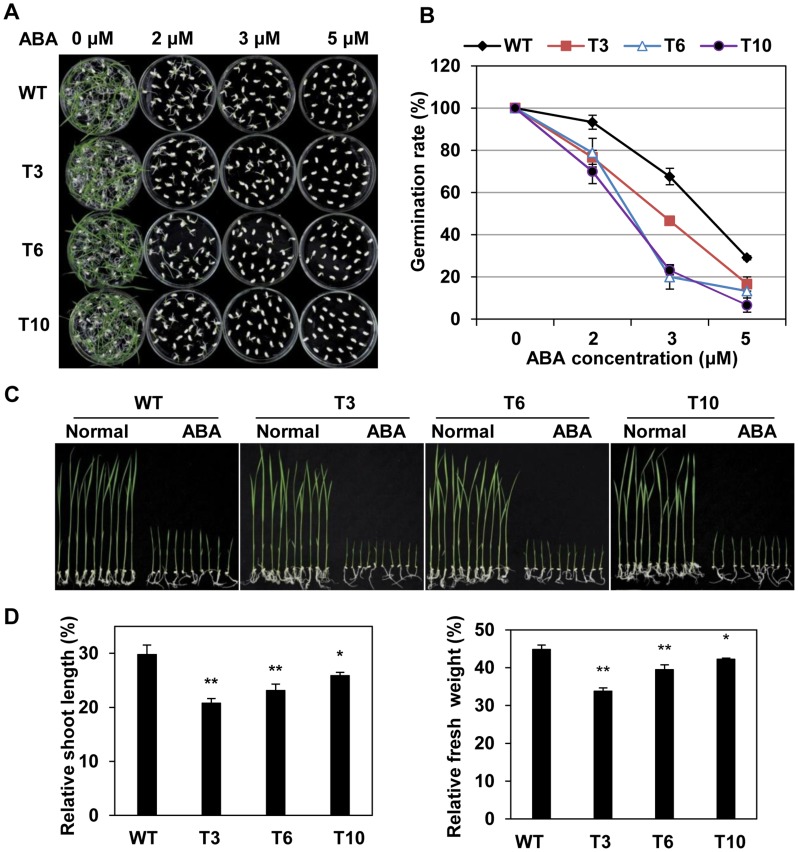
ABA hypersensitivity of *OsMYB48-1* overexpression plants. (A) Germination performance of *OsMYB48-1-*overexpression and WT seeds on 1/2 MS medium containing 0, 2, 3, or 5 μM/L ABA measured at 10 d after initiation. (B) Calculation of the germination rates of transgenic and WT seeds. Values are means ± SE (n = 3). (C) Performance of the *OsMYB48-1* overexpression plants and WT in 1/2 MS medium containing 3 μM/L ABA. (D) The relative shoot length and fresh weight of the transgenic and WT plants grown on normal and ABA-containing medium were compared. SE based on 30 seedlings. Values are means ± SE. * and ** indicate significant difference at P<0.05 and P<0.01 probability, respectively.

Since the expression of *OsMYB48-1* was induced by ABA, we had investigated the exogenous ABA sensitivity of transgenic plants at germination stage. Results showed non-significant differences in germination rates between the overexpression lines and WT at 0 μM ABA, however, it was significantly lower in the transgenics as compared to WT at 2, 3 and 5 μM ABA (Fig. 6A, B). The results suggested that *OsMYB48-1* overexpression plants were hypersensitive to ABA than WT at the germination stage.

Hypersensitivity response of *OsMYB48-1* overexpression plants to exogenous ABA was also investigated at the post-germination stage. *OsMYB48-1-*overexpression lines had significantly (P<0.05) more suppression of fresh shoot growth (20.78% to 25.87% of normal growth) and fresh weight growth (33.75% to 42.21% of normal growth) than that of WT (29.78% and 44.84% of the normal growth, respectively) on the ABA treatments (Fig. 6C, D). Overexpression of *OsMYB48-1* could increase exogenous ABA sensitivity at post-germination stages as observed by the results.

### Endogenous ABA Accumulation and the Expression of ABA-related Genes in *OsMYB48-1*overexpression Plants

**Figure 7:**
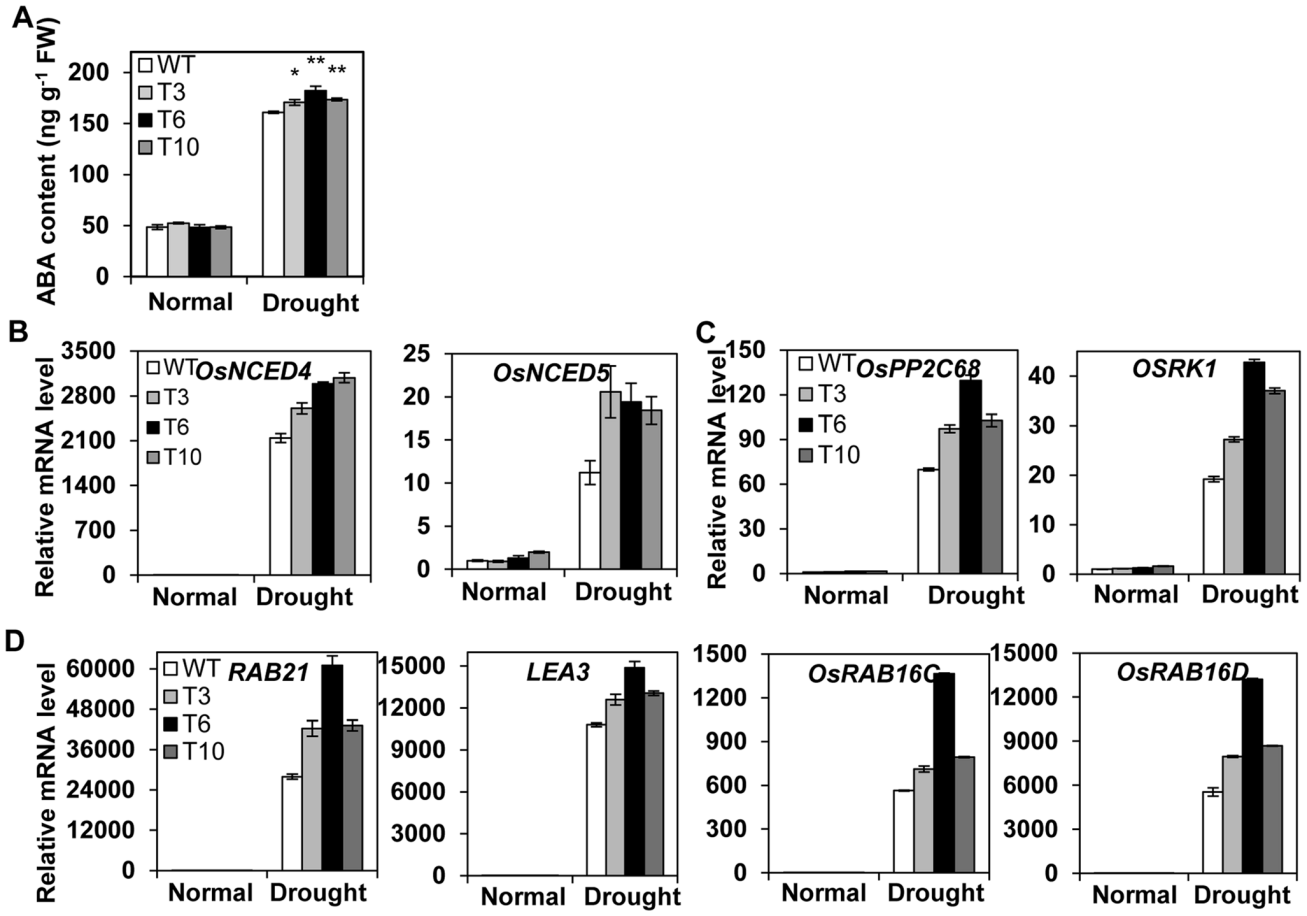
ABA accumulation and the expression of ABA -related genes under drought stress. (A) ABA contents in normal or drought treated seedlings. Data are means SE (n = 4). (B) qRT-PCR analysis of the expression of ABA biosynthesis genes. (C) Transcript levels of ABA early signaling genes. (D) Transcript levels of ABA later responsive genes under normal or drought stress conditions (withholding water for 5 d). Values are means ± SE (n = 3).

Enhanced drought tolerance and hypersensitive response of *OsMYB48-1* overexpression plants to exogenous ABA application necessitates the determination of endogenous ABA content both in the transgenic and WT plants. Transgenic and WT plants showed non-significant differences for endogenous ABA content under normal growth conditions, however, it was significantly higher in transgenic seedlings (170–182 ng/g) than the WT (161 ng/g) (Fig. 7A), indicating the role of *OsMYB48-1* in regulating ABA synthesis under drought stress conditions.

Since the transgenic plants were sensitive to exogenous ABA and accumulated more endogenous ABA under drought stress conditions, the transcription levels of some ABA biosynthesis and signaling genes were analyzed. As shown in Figure 7B, the transcript levels of two ABA biosynthesis genes, Os*NCED4* and Os*NCED5* in both the transgenic plants and WT were clearly up-regulated by drought stress, while the expression levels of these genes were clearly higher in *OsMYB48-1* overexpression lines as compared to WT. As shown in Fig. 7C, two early ABA signaling genes, *OsPP2C68* and *OSRK1*, showed much higher expression levels in transgenic plants than that in WT under drought stress conditions. The expression levels of four well-documented ABA late responsive genes, *RAB21*, *OsLEA3*, *RAB16C* and *RAB16D*, showed non-significant differences between the transgenic plants and WT under normal growth conditions. However, under drought stress conditions significant differences were observed for higher expression levels of these genes in transgenic plants as compared to WT were detected (Fig. 7D). Together, these data suggested that *OsMYB48-1* play an important role in drought stress-induced ABA-biosynthesis through regulating the expression of ABA-biosynthesis genes, such as *OsNCED4* and *OsNCED5*, consequently, regulating early ABA signaling genes, such as *OsPP2C68* and *OSRK1*, and late responsive genes, such as *RAB21*, *OsLEA3*, *RAB16C* and *RAB16D*.

## Discussion

### *OsMYB48-1*, a Novel MYB-related TF of Rice in Abiotic Stress Tolerance

The identification of some MYB TFs in rice and their roles in abiotic stress response has been reported recently [20], [22], [34], however, our results indicated that *OsMYB48-1* to have unique features as compared to other MYB members in rice.

The expression of *OsMYB48-1* was found to be tissue specific that showed highest expression levels in roots at both seedling and reproductive stages, followed by leaves and sheath at reproductive stages. However, *OsMYB*2 was detected to have a greatest expression levels in leaves, followed by roots and shoots [22]. The highest level of transcripts of *OsMYB3R-2* was found in young stems while the lowest was in spikes [34]. Differential tissue expression patterns of *OsMYB48-1* in comparison to other MYB genes involved in abiotic stress tolerance indicates a possibility of different role for it.

The expression profiles of *OsMYB48-1* under abiotic stress treatments were much different from those of *Osmyb4*, *OsMYB2,* and *OsMYB3R-2*, which were reported to be involved in drought tolerance in rice. In contrast, the expression of *OsMYB2* was significantly induced by salt, cold, and PEG treatments [22]. *OsMYB3R-2* was induced by cold, drought, and salt stress [35]. The expression of *Osmyb4* in rice was only induced by cold but not by other stresses [20]. Our results showed that the expression of *OsMYB48-1* was strongly induced by PEG, H_2_O_2_, ABA, and dehydration treatments, while slightly induced by salt and cold treatments. Therefore, based on the specific stress inductive patterns of *OsMYB48-1* as compared to other MYB proteins suggest it to play a special role in response to abiotic stresses in rice.

Moreover, four single MYB-like domain proteins in rice have been functionally studied. *OsMYBS1*, *OsMYBS2* and *OsMYBS3* mediate α-amylase gene expression [36], and *OsMYBS3* plays a role in cold tolerance in rice [25]. *OsAID1* involves in spikelet sterility [37]. To our knowledge, it is the first report that MYB-related type TF, *OsMYB48-1*, involved in drought and salt tolerance in rice. Placing all the results together we could deduce OsMYB48-1 to be a novel MYB-related TF of rice for abiotic stress tolerance.

### Positive Effect of *OsMYB48-1* in Drought and Salt Stress Tolerance

Growth retardation has been frequently observed in rice plants overexpressing stress-responsive TFs, such as *OsDREB1A*, *OsDREB1B, OsWRKY89* and *OsNAC6* in rice [38]–[40]. However, the overexpression plants of *OsMYB48-1* showed no obvious phenotypic changes from that of the WT under normal growth conditions. Overexpression of *OsMYB48-1* in rice resulted in increased sensitivity to exogenous ABA at both germination and post-germination stages and accumulated more endogenous ABA content under drought stress conditions, suggesting *OsMYB48-1* to possibly play an important role in ABA-signaling transduction pathway. It has been widely accepted that a positive regulator of ABA signaling could play a positive role in the stress tolerance of plants. Consequently, transgenic plants overexpressing *OsMYB48-1* showed improved growth performance under simulated drought stress conditions created by mannitol and PEG treatments, and also under dry soil conditions with enhanced survival rate. Interestingly, *OsMYB48-1* was only slightly up-regulated by high salinity, overexpression plants showed tolerance to salt stress, which suggested that *OsMYB48-1* may be involved in salt stress tolerance through a cross-talk between drought and high salinity stress response pathway. Overall, *OsMYB48-1* overexpression plants demonstrated improved drought and salt tolerance besides possessing an unaltered phenotype under normal conditions in comparison to WT implies the usefulness of *OsMYB48-1* in genetic improvement of abiotic stress tolerance in rice.

### Possible Mechanisms for the Drought Tolerance of *OsMYB48-1* Overexpression Plants

ABA plays a key role in abiotic stress response. In response to abiotic stresses, transgenic plants overexpressing some ABA biosynthesis-related genes increased ABA content dramatically to cope with the stress, such as transgenic cottons overexpressing *AtLOS5* [41], transgenic *Arabidopsis* overexpressing *OsNCED3* [42]. Here, we identified that the expression of *OsMYB48-1* could be induced by exogenous ABA, and *OsMYB48-1* overexpression plants showed hypersensitivity to exogenous ABA. Accumulation of the endogenous ABA content in transgenic plants was higher than in WT to confer drought tolerance, implicating *OsMYB48-1* possible role in stress-induced ABA accumulation. The expression of 9-*cis* epoxycarotenoid dioxygenase (*NCED*) is generally considered to be the rate-limiting step among all the steps of drought stress-induced ABA biosynthesis pathway [43]. Expression levels of rice *NCED* genes in transgenic plants and WT under normal and drought stress conditions were comprehensively studied by us. Results showed similar low expression levels of *OsNCED4* and *OsNCED5* in transgenic plants and WT under normal conditions which suggested *OsMYB48-1* alone may not be sufficient to regulate the ABA biosynthesis. However, the high expression levels of *OsNCED4* and *OsNCED5* in transgenic plants under drought stress conditions clearly suggested that *OsMYB48-1* possible role in stress-induced ABA-biosynthesis along with additional factors.

The increased endogenous ABA levels in response to drought stress results in further modifying the expression of many stress-responsive genes [41], we also detected the expression levels of some early ABA signaling genes and late ABA-responsive genes in the transgenic plants and WT under normal and drought stress conditions. OsPP2C68 encodes a protein phosphatase 2C, which plays a prime role in ABA-mediated signaling network related to stress responses [44]. OSRK1 encodes a protein kinase of SnRK2 family, which is associated with ABA signaling [45]. Under drought stress conditions, *OsPP2C68* and *OSRK1* had higher expression levels in the transgenic plants than that in WT. Four LEA genes, *RAB21*, *OsLEA3*, *RAB16C* and *RAB16D* have been reported to be marker genes in ABA-dependent stress response way [12], [46], [47]. We identified upon drought stress, the expression levels of these genes were significantly higher in the transgenic plants than WT. Overall the results suggested that the overexpression of *OsMYB48-1* had further induced ABA synthesis in transgenic plants under drought stress conditions, thus, leading to increased expression levels of ABA signaling and –responsive genes.

Proline acts as one of the most common osmolytes in plants. The step catalyzed by enzyme pyrroline-5-carboxylate synthase (P5CS) is the key step in proline synthesis [48]. Overexpression of a *P5CS* gene in tobacco (*Nicotiana tabacum*) demonstrated the causal relationship between proline synthesis and tolerance of drought and salt stress [49]. A greater accumulation of free proline was found in the *OsMYB48-1* overexpression plants than that in WT under drought stress conditions. Meanwhile, higher expression levels of *OsP5CS1* and *OsP5CS2* were detected in the transgenic plants as compared to WT under drought stress conditions. These results suggested that the accumulated proline might be seen as a response of *OsMYB48-1* transgenic plants towards abiotic stresses.

Furthermore, the transgenic plants overexpressing drought-tolerance related genes showed slower rate of water loss under dehydration conditions, as observed in transgenic Arabidopsis overexpressing *TaMYB30-B* [50] and *OsMYB3R-2* [34] and transgenic rice overexpressing *OsbZIP23* [12] and *OsMIOX* [27]. Based on the earlier reports, overexpression of *OsMYB48-1* transgenic rice had showed lower rate of water loss and improved drought tolerance in comparison to WT indicating the vital role of *OsMYB48-1* in protecting water loss especially under drought stress conditions.

**Figure 8:**
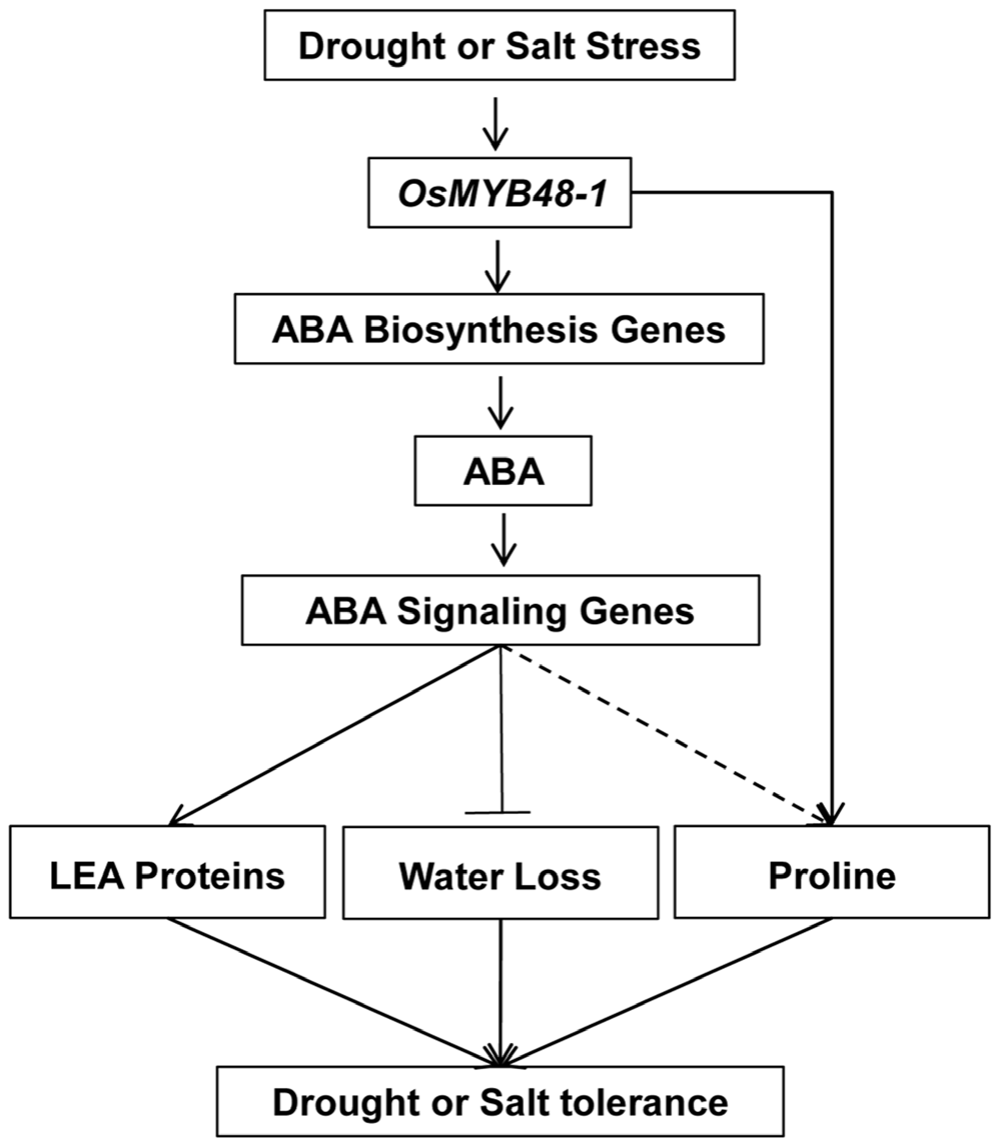
Proposed model depicting the function of *OsMYB48-1* in the regulation of drought and salinity stress tolerance.

We tried to summarize all the results obtained into a model to explain the potential role of *OsMYB48-1* in regulating drought stress tolerance in rice (Fig. 8). In conclusion, this study isolated and characterized a novel MYB-related type TF *OsMYB48-1*, which regulates the expression of ABA synthesis genes thus leading to increased endogenous ABA accumulation under drought stress conditions. Moreover, overexpression of *OsMYB48-1* increased LEA protein and proline content while reduced rate of water loss in transgenic plants to confer drought tolerance. In addition, the overexpression of *OsMYB48-1* did not alter the phenotypes of the transgenic plants carrying them. Therefore, we like to conclude that *OsMYB48-1* may be an effective gene for improving drought and salinity tolerance in rice.

## Supporting Information

Figure S1 **Protein sequence analysis of OsMYB48-1. (A)** Phylogenetic tree of the MYB members in Arabidopsis and rice. The phylogenetic tree was constructed in MEGA5 software based on neighbor-joining method. Numbers indicate percentage values after 1000 replications. **(B)** Multiple sequence alignment of OsMYB48-1, OsMYB146, OsMYB154, AtMYB48, AtMYB59 with DNAMAN software. Conserved SANT DNA-binding domain is indicated by red boxes.
(TIF)

Figure S2 **RNA interference of** ***OsMYB48-1*** **. (A)** RNAi construct of *OsMYB48-1* for rice transformation. **(B)** Expression level of *OsMYB48-1* in RNAi transgenic lines analyzed by qRT-PCR.
(TIF)

Figure S3 **Overexpression of** ***OsMYB48-1*** **. (A)** Overexpression construct of *OsMYB48-1* for rice transformation. **(B)** The expression level of *OsMYB48-1* in WT and overexpression transgenic lines analyzed by qRT-PCR. Transgenic lines T3, T6, T10 showed the highest expression level, were selected for further study.
(TIF)

Table S1 **Primer sequences used in this study.**
(DOCX)

Table S2 **Potential stress-related** ***cis*** **-elements in the promoter of** ***OsMYB48-1*** **.**
(DOCX)
