# Supplementary figures and images for: Overexpression of OsMYB48-1, a Novel MYB-Related Transcription Factor, Enhances Drought and Salinity Tolerance in Rice
Source: PLoS One. 2014 Mar 25;9(3):e92913. doi: 10.1371/journal.pone.0092913 (PMC3965499; doi:10.1371/journal.pone.0092913)

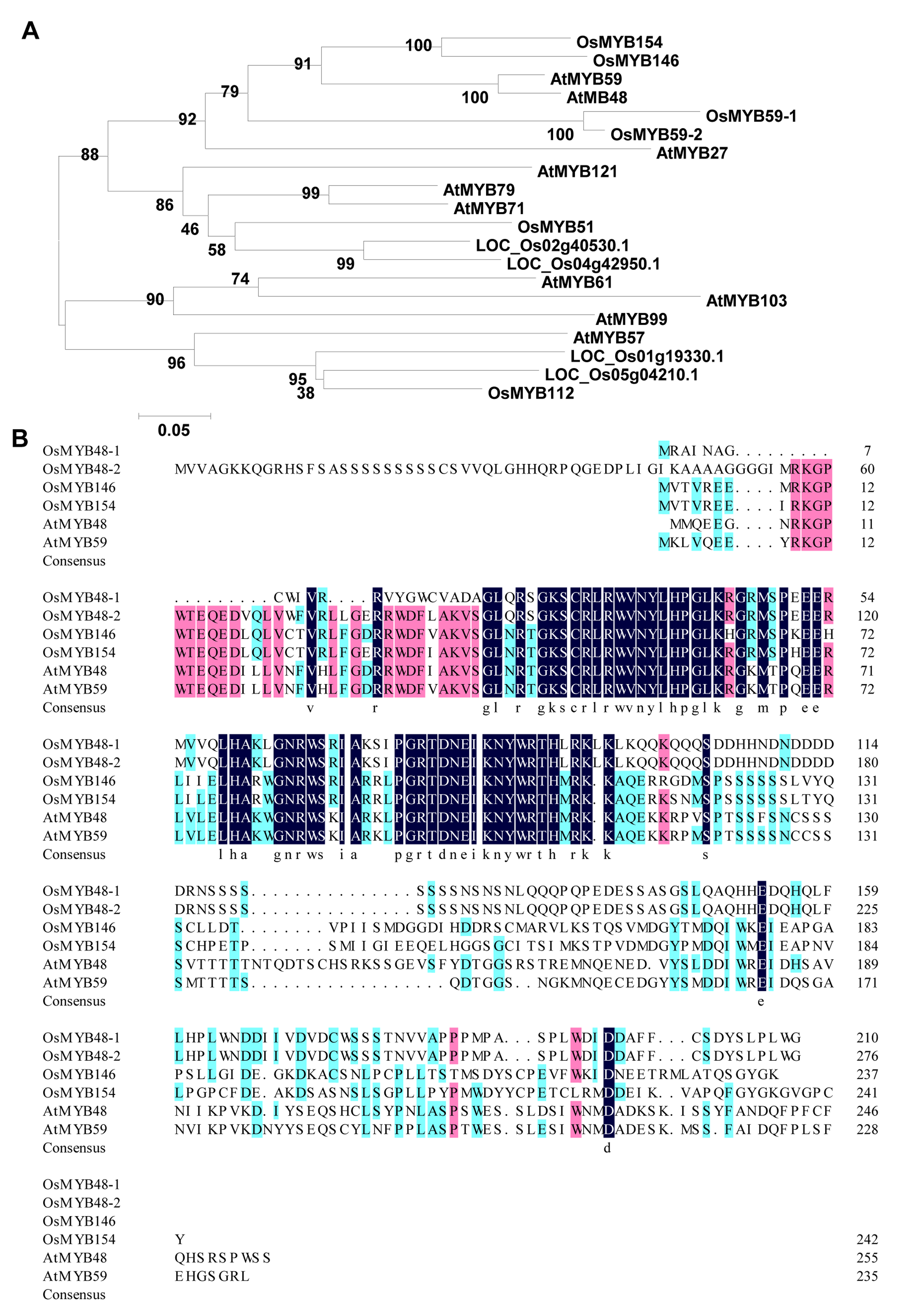

Supplement: Figure S1 — Protein sequence analysis of OsMYB48-1. (A) Phylogenetic tree of the MYB members in Arabidopsis and rice. The phylogenetic tree was constructed in MEGA5 software based on neighbor-joining method. Numbers indicate percentage values after 1000 replications. (B) Multiple sequence alignment of OsMYB48-1, OsMYB146, OsMYB154, AtMYB48, AtMYB59 with DNAMAN software. Conserved SANT DNA-binding domain is indicated by red boxes. (TIF) [file pone.0092913.s001.tif]

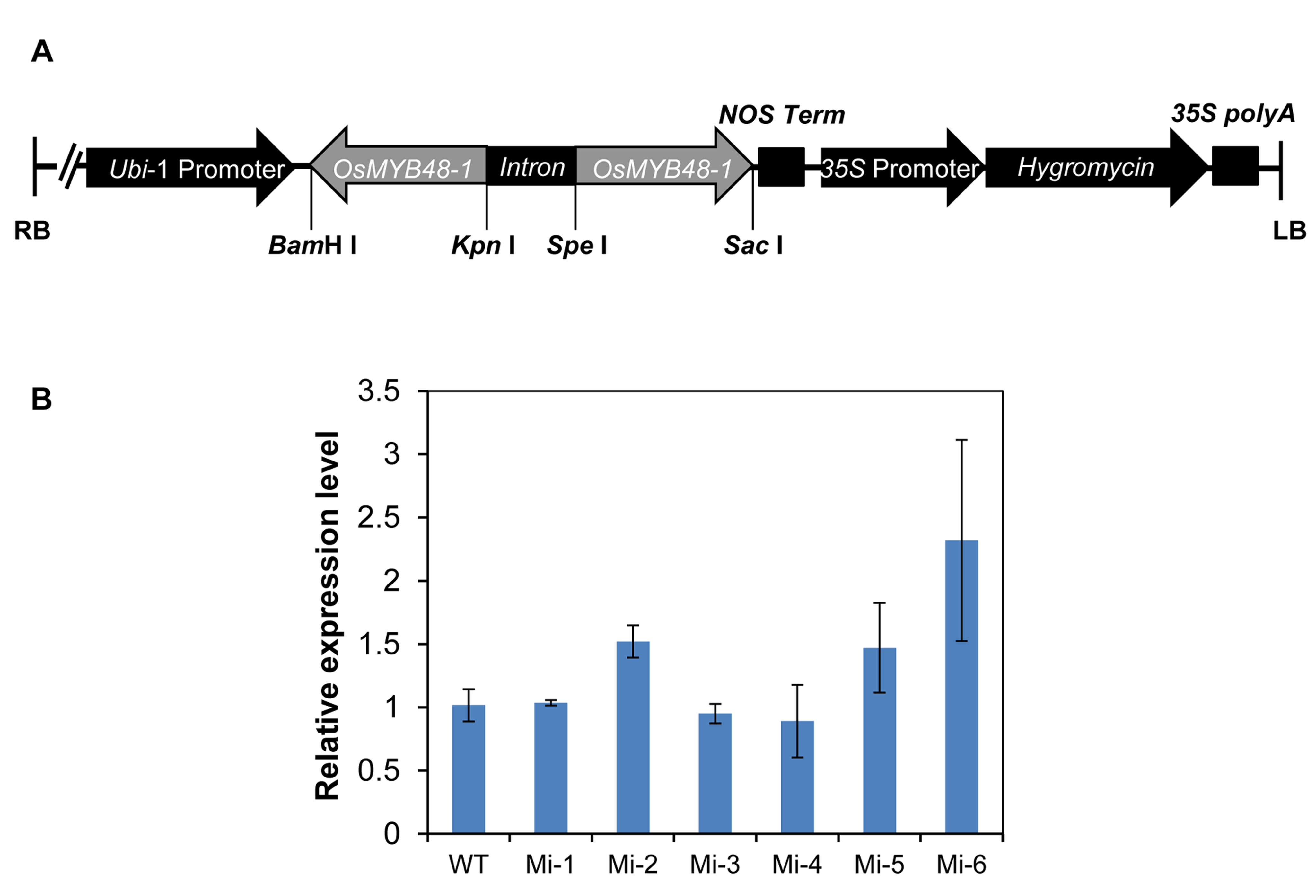

Supplement: Figure S2 — RNA interference of OsMYB48-1 . (A) RNAi construct of OsMYB48-1 for rice transformation. (B) Expression level of OsMYB48-1 in RNAi transgenic lines analyzed by qRT-PCR. (TIF) [file pone.0092913.s002.tif]

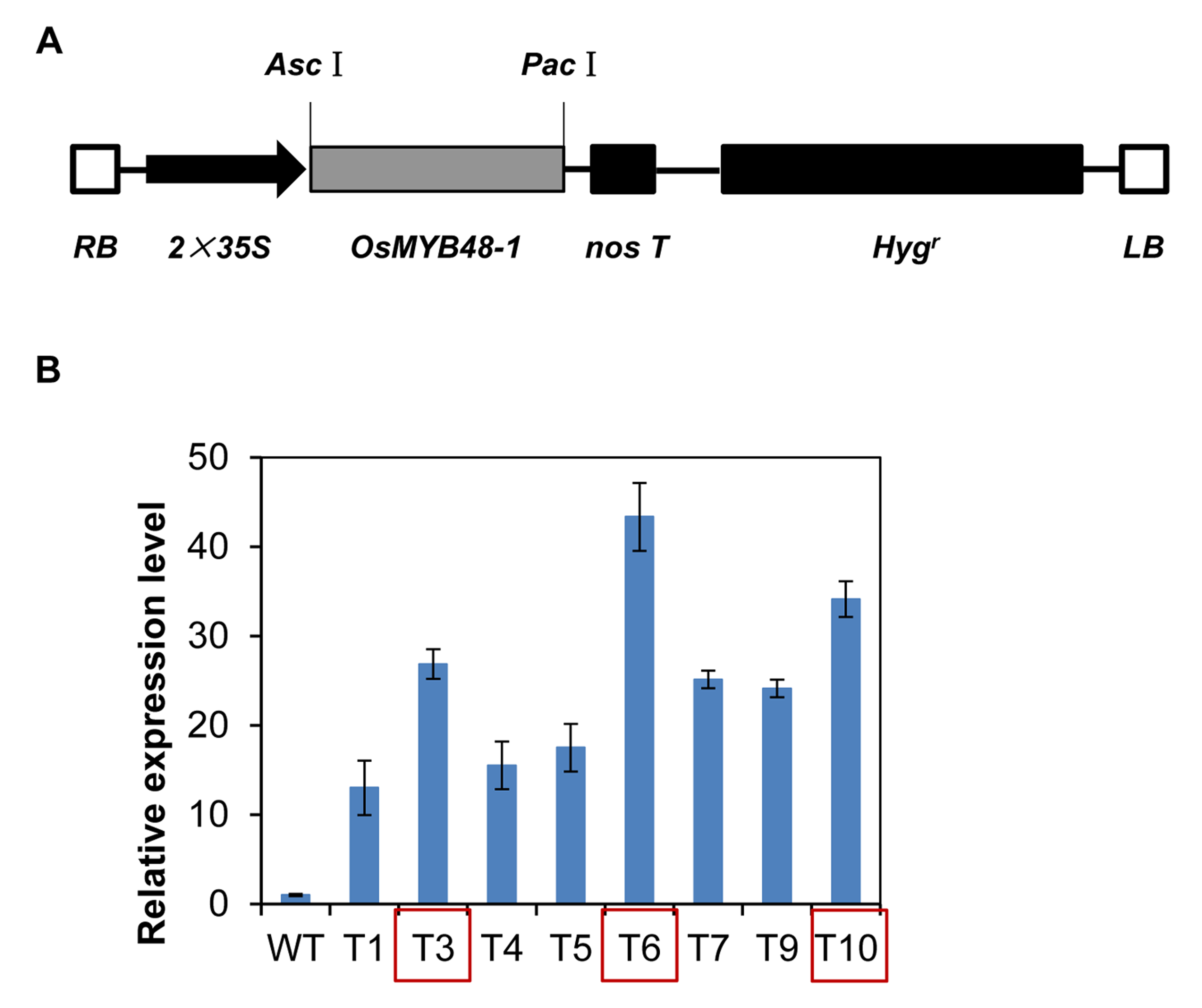

Supplement: Figure S3 — Overexpression of OsMYB48-1 . (A) Overexpression construct of OsMYB48-1 for rice transformation. (B) The expression level of OsMYB48-1 in WT and overexpression transgenic lines analyzed by qRT-PCR. Transgenic lines T3, T6, T10 showed the highest expression level, were selected for further study. (TIF) [file pone.0092913.s003.tif]
