# Supplementary material for: Overexpression of OsMYB48-1, a Novel MYB-Related Transcription Factor, Enhances Drought and Salinity Tolerance in Rice
Source: PLoS One. 2014 Mar 25;9(3):e92913. doi: 10.1371/journal.pone.0092913 (PMC3965499; doi:10.1371/journal.pone.0092913)
Supplement: Table S1 — Primer sequences used in this study. (DOCX) [file pone.0092913.s004.docx]

**Table S1. Primer sequences used in this study.**

| Primer name | Sense primer (5'->3') | Anti-sense primer (5'->3') | Prodction size (bp) |
| --- | --- | --- | --- |
| *RT-PCR* | | | |
| *OsMYB48* | AGCAAAGGTGTCAGGTTTGCA | GGTAGCGAATAATCCGAGCAGAAG | 778 (*OsMYB48-1*)  567 (*OsMYB48-2*) |
| *Actin* | ATTTGGCACCACACATTCTAC | ATAACCTTCGTAGATTGGGACT | 255 |
| *(b) qRT-PCR* | | | |
| *OsMYB48-1* | TGGGATTTCTTAGCAAAGGTGTC | ACACACCACCCATACACACGAC | 225 |
| *Actin* | ATTTGGCACCACACATTCTAC | ATAACCTTCGTAGATTGGGACT | 255 |
| *PP2C* | CGCAGCTCCGACAACATCT | GCTGGGTGACACTCTCTCTACAAG | 77 |
| *OSRK1* | AGTACACCAAGCAGGTGAAGCA | GCAACAGCAAAGCTTGAACTCA | 86 |
| *LEA3* | GCCGTGAATGATTTCCCTTTG | CACACCCGTCAGAAATCCTCC | 148 |
| *RAB21* | CACACCACAGCAAGAGCTAAGTG | TGGTGCTCCATCCTGCTTAAG | 101 |
| *RAB16C* | TTCCCGGCCAGCACTAAAT | AAACTGCACGTACATCACGACAT | 67 |
| *RABl6D* | CGGGTAAACAATAAAGTCGTGATG | GCGCACTTACATACAGTGCTACGT | 74 |
| *OsNCED4* | GATTGCACGGCACCTTCATT | CTCTGTAATTTGATTTTTCACTGGCTAAT | 88 |
| *OsNCED5* | GGATGGGCTGAACTTCTTCCAG | CAGCACATTCGTGATGAACCCT | 79 |
| *OsP5CS1* | CCCGTCCCGGAGCTTCGTGAG | CCTAAGTCGCTGTCGCCCCAC | 199 |
| *OsP5CS2* | GCTGCCGTCGGTCAGAGTG | CTCGTATGGTTGCCTCCTGGT | 216 |
| *(c) Plasmid construction* | |  |  |
| *M-OE* | TTGGCGCGCCATGCGTGCGATCAACGCT | CCTTAATTAACTATCCCCAGAGAGGTAGCGAA | 653 |
| *M-RNAi* | GGGGTACCACTAGTACCAACTGTTCCTTCATCCT | GGGGATCCGAGCTCATATGGATGTGTACGCAGCTA | 490 |
| *M-GFP* | CCTTAATTAATGCGTGCGATCAACGCT | TTGGCGCGCCAGAGAGGTAGCGAATAATCCG | 644 |
| *M-FL* | CCGGAATTCATGCGTGCGATCAACGCT | CGCGGATCCCCTATCCCCAGAGAGGTAGCGAA | 652 |
| *M-dC1* | CCGGAATTCAAGCGAGGGAGGATGAGCC | CGCGGATCCCCTATCCCCAGAGAGGTAGCGAA | 523 |
| *M-dC2* | CCGGAATTCTCCAACAGCAACAGCAACCT | CGCGGATCCCCTATCCCCAGAGAGGTAGCGAA | 280 |
| *M-dC3* | CCGGAATTCCAGCACCAACTGTTCCTTCA | CGCGGATCCCCTATCCCCAGAGAGGTAGCGAA | 190 |
| *M-dC4* | CCGGAATTCTGGAGCAGCAGCACCAAC | CGCGGATCCCCTATCCCCAGAGAGGTAGCGAA | 130 |
| *MdC5* | CCGGAATTCATGCGTGCGATCAACGCT | CGCGGATCCCGGAGGACGAAGAGGAGGAGG | 388 |
