# Supplementary material for: Overexpression of OsMYB48-1, a Novel MYB-Related Transcription Factor, Enhances Drought and Salinity Tolerance in Rice
Source: PLoS One. 2014 Mar 25;9(3):e92913. doi: 10.1371/journal.pone.0092913 (PMC3965499; doi:10.1371/journal.pone.0092913)
Supplement: Table S2 — Potential stress-related cis -elements in the promoter of OsMYB48-1 . (DOCX) [file pone.0092913.s005.docx]

**Table S2. Potential stress-related *cis*-acting elements in the promoters of *OsMYB48-1*.**

| Cis-acting elements | Sequence | Copy number | Function | References |
| --- | --- | --- | --- | --- |

| W-box | TTGAC, TGACT, TGAC, TGAC, TTTGACY | 36 | Involved in activation of genes involved in response to wounding and defense | [[1](#_ENREF_1),[2](#_ENREF_2)] |
| --- | --- | --- | --- | --- |
| MYC | CATGTG,CACATG, CANNTG | 18 | Involved in drought- and ABA-regulated gene expression. | [[3-6](#_ENREF_3)] |
| MYB | ACCWWCC, WAACCA, GTTAGTT,YAACKG, CNGTTR, AACGG, MACCWAMC, CCWACC, GGATA | 18 | Involved in regulation of drought inducible gene expression | [[4-6](#_ENREF_4)] |
| CURECORECR | GTAC | 16 | Involved in oxygen-response | [[7](#_ENREF_7),[8](#_ENREF_8)] |
| BIHD1OS | TGTCA | 8 | Involved in disease resistance responses. | [[9](#_ENREF_9)] |
| Erd1 | ACGT | 6 | Required for early response to dehydration | [[10](#_ENREF_10)] |
| DPBFCOREDCDC3 | ACACNNG | 6 | Involved in ABA response | [[11-13](#_ENREF_11)] |
| RAV | CAACA | 4 | RAV1 protein recognition sequence | [[14](#_ENREF_14)] |
| CGCG box | VCGCGB | 4 | Involved in ethylene signaling, abscisic acid signaling, and light signal perception | [[15](#_ENREF_15)] |
| [GCC-box](http://bioinfo.cau.edu.cn/ProFITS/BS_anno.php?source=PLACE&BS=GCCCORE) | GCCGCC | 3 | Ethylene-responsive element | [[16](#_ENREF_16),[17](#_ENREF_17)] |
| LTRECORE | CCGAC | 2 | Core of low temperature responsive element, involved in cold, drought and ABA responsiveness | [[18](#_ENREF_18),[19](#_ENREF_19)] |
| DRE/CRT | RCCGAC | 2 | Function in drought-, high-salt- and cold-responsive | [[20](#_ENREF_20),[21](#_ENREF_21)] |
| CBF | RYCGAC | 2 | Dehydration-responsive element (DRE) binding proteins (DREBs) | [[22](#_ENREF_22)] |
| ERE | AWTTCAAA | 1 | Ethylene responsive element | [[23](#_ENREF_23),[24](#_ENREF_24)] |

**Supplemental reference**

1. Eulgem T, Rushton PJ, Robatzek S, Somssich IE (2000) The WRKY superfamily of plant transcription factors. Trends in Plant Science 5: 199-206.

2. Maleck K, Levine A, Eulgem T, Morgan A, Schmid J, et al. (2000) The transcriptome of *Arabidopsis thaliana* during systemic acquired resistance. Nat Genet 26: 403-310.

3. Abe H, Yamaguchi-Shinozaki K, Urao T, Iwasaki T, Hosokawa D, et al. (1997) Role of arabidopsis MYC and MYB homologs in drought- and abscisic acid-regulated gene expression. Plant Cell 9: 1859-1868.

4. Abe H, Urao T, Ito T, Seki M, Shinozaki K, et al. (2003) Arabidopsis AtMYC2 (bHLH) and AtMYB2 (MYB) function as transcriptional activators in abscisic acid signaling. The Plant Cell Online 15: 63-78.

5. Maeda K, Kimura S, Demura T, Takeda J, Ozeki Y (2005) DcMYB1 acts as a transcriptional activator of the carrot phenylalanine ammonia-lyase gene (*DcPAL1*) in response to elicitor treatment, UV-B irradiation and the dilution effect. Plant Mol Biol 59: 739-752.

6. Shinozaki K, Yamaguchi-Shinozaki K (2000) Molecular responses to dehydration and low temperature: differences and cross-talk between two stress signaling pathways. Curr Opin Plant Biol 3: 217-223.

7. Quinn JM (2000) Coordinate copper- and oxygen-responsive *Cyc6* and *Cpx1* expression in *Chlamydomonas* is mediated by the same element. Journal of Biological Chemistry 275: 6080-6089.

8. Quinn JM, Eriksson M, Moseley JL, Merchant S (2002) Oxygen deficiency responsive gene expression in Chlamydomonas reinhardtii through a copper-sensing signal transduction pathway. Plant Physiol 128: 463-471.

9. Luo H, Song F, Goodman RM, Zheng Z (2005) Up-regulation of *OsBIHD1*, a rice gene encoding BELL homeodomain transcriptional factor, in disease resistance responses. Plant Biol (Stuttg) 7: 459-468.

10. Simpson SD, Nakashima K, Narusaka Y, Seki M, Shinozaki K, et al. (2003) Two different novel *cis*-acting elements of *erd1*, a *clpA* homologous *Arabidopsis* gene function in induction by dehydration stress and dark-induced senescence. The Plant Journal 33: 259-270.

11. Finkelstein RR, Lynch TJ (2000) The Arabidopsis abscisic acid response gene *ABI5* encodes a basic leucine zipper transcription factor. The Plant Cell Online 12: 599-609.

12. Kim SY, Chung H-J, Thomas TL (1997) Isolation of a novel class of bZIP transcription factors that interact with ABA-responsive and embryo-specification elements in the *Dc3* promoter using a modified yeast one-hybrid system. The Plant Journal 11: 1237-1251.

13. Lopez-Molina L, Chua N-H (2000) A null mutation in a bZIP factor confers ABA-insensitivity in *Arabidopsis thaliana*. Plant and Cell Physiology 41: 541-547.

14. Kagaya Y, Ohmiya K, Hattori T (1999) RAV1, a novel DNA-binding protein, binds to bipartite recognition sequence through two distinct DNA-binding domains uniquely found in higher plants. Nucleic Acids Research 27: 470-478.

15. Yang T, Poovaiah BW (2002) A calmodulin-binding/CGCG box DNA-binding protein family involved in multiple signaling pathways in plants. J Biol Chem 277: 45049-45058.

16. Brown RL, Kazan K, McGrath KC, Maclean DJ, Manners JM (2003) A role for the GCC-box in jasmonate-mediated activation of the *PDF1.2* gene of Arabidopsis. Plant Physiol 132: 1020-1032.

17. Chakravarthy S, Tuori RP, D'Ascenzo MD, Fobert PR, Despres C, et al. (2003) The tomato transcription factor Pti4 regulates defense-related gene expression via GCC box and non-GCC box *cis* elements. Plant Cell 15: 3033-3050.

18. Baker S, Wilhelm K, Thomashow M (1994) The 5'-region of *Arabidopsis thaliana cor15a* has *cis*-acting elements that confer cold-, drought- and ABA-regulated gene expression. Plant Molecular Biology 24: 701-713.

19. Kim H-J, Kim Y-K, Park J-Y, Kim J (2002) Light signalling mediated by phytochrome plays an important role in cold-induced gene expression through the C-repeat/dehydration responsive element (C/DRE) in *Arabidopsis thaliana*. The Plant Journal 29: 693-704.

20. Dubouzet JG, Sakuma Y, Ito Y, Kasuga M, Dubouzet EG, et al. (2003) *OsDREB* genes in rice, *Oryza sativa* L., encode transcription activators that function in drought-, high-salt- and cold-responsive gene expression. The Plant Journal 33: 751-763.

21. Qin F, Sakuma Y, Li J, Liu Q, Li Y-Q, et al. (2004) Cloning and functional analysis of a novel DREB1/CBF transcription factor involved in Cold-Responsive gene expression in *Zea mays* L. Plant and Cell Physiology 45: 1042-1052.

22. Xue GP (2002) Characterisation of the DNA-binding profile of barley HvCBF1 using an enzymatic method for rapid, quantitative and high-throughput analysis of the DNA-binding activity. Nucleic Acids Research 30: e77.

23. Itzhaki H, Maxson JM, Woodson WR (1994) An ethylene-responsive enhancer element is involved in the senescence-related expression of the carnation glutathione-S-transferase (*GST1*) gene. Proceedings of the National Academy of Sciences 91: 8925-8929.

24. Montgomery J, Goldman S, Deikman J, Margossian L, Fischer RL (1993) Identification of an ethylene-responsive region in the promoter of a fruit ripening gene. Proceedings of the National Academy of Sciences 90: 5939-5943.
